# Plain water consumption is associated with lower intake of caloric beverage: cross-sectional study in Mexican adults with low socioeconomic status

**DOI:** 10.1186/s12889-015-1699-0

**Published:** 2015-04-19

**Authors:** Daniel Illescas-Zarate, Juan Espinosa-Montero, Mario Flores, Simon Barquera

**Affiliations:** Departamento de Nutricion y Bioprogramacion, Instituto Nacional de Perinatologia, Montes Urales 800, CP 11000 DF Mexico, Mexico; Centro de Investigación en Nutrición y Salud, Instituto Nacional de Salud Publica, Av. Universidad 655, CP 62100 Cuernavaca, Mexico

**Keywords:** Plain water, Caloric beverages, Sweetened beverages, Adults, Diet

## Abstract

**Background:**

Plain water (PW) should be the main beverage consumed by the population. However, consumption of caloric beverages (CB) has increased considerably worldwide. The purpose of this paper is to analyze the association between CB and PW intake in Mexican adults with a low socioeconomic status (SES).

**Methods:**

In a cross-sectional design, beverage consumption was evaluated with a 24-h beverages recall using the five-step multiple-pass method recommended by the U.S. Department of Agriculture. Physical activity, anthropometric and sociodemographic information were obtained. CB was defined as those beverages that provide energy, with the exception of low-fat milk and beverages with noncaloric sweeteners. Participants were classified into five groups according to their PW consumption (nondrinkers and four quartiles). Differences between groups were evaluated with ANOVA and Bonferroni tests for multiple comparisons among quartiles. A two-stage Heckman regression model was designed with robust standard errors, adjusting for potential confounders.

**Results:**

A total of 1108 adults between 21 and 59 years of age were evaluated. A negative association was noted between PW intake and CB consumption (p <0.001) with the exception of natural juice, which was positive (p <0.01) and sodas that no differences were found between quartiles. Specifically, for every milliliter of PW, the intake of CB was 3.4, 1.3, 0.68 and 0.38 mL in each quartile, respectively (p <0.001). In Heckman’s model, PW consumers were 0.5 times less likely to consume CB (p = 0.029). This probability increased to 0.9 for low-fat milk, skim milk and beverages without added sugar (LFM-BWAS) consumers (p <0.001). Also, for every 100 mL of PW consumption, CB intake diminished by 20 mL (p <0.001). In turn, for every 100 mL of LFM-BWAS consumption, a reduction of 47 mL in CB was observed (p <0.001).

**Conclusions:**

Higher PW consumption was associated with lower CB consumption. This association suggests that future studies are warranted to determine if increasing PW intake in a low SES Mexican population can reduce intake of CB.

**Electronic supplementary material:**

The online version of this article (doi:10.1186/s12889-015-1699-0) contains supplementary material, which is available to authorized users.

## Background

Plain water (PW) should be the main beverage consumed by the population because it is necessary for maintenance of adequate hydration for various vital cellular processes as well as providing no calories [1]. Consumption of caloric beverages (CB), particularly those with added sugar, has increased significantly worldwide [2].

Daily intake of CB per capita by Mexican adults in 2006 was estimated to be 772 mL, contributing 21.7% to the total energy per day (403 kcal). It was observed that the proportion of households purchasing sodas increased from 48 to 60% in 18 years (1989–2006) [3] and energy from beverages like fresh fruit beverages (common name “agua fresca”), coffee/tea and milk with added sugar have also increased from 1999 to 2012 [4]. Multiple studies have shown that CB consumption is strongly associated with weight gain, type 2 diabetes mellitus and cardiovascular diseases [5-9]. On the other hand, the intake of PW does not have any adverse effects on people’s health, improves hydration [10], increases satiety [11] and thermogenesis [12], contributes to a minor energy intake of a total diet [13] and it has been observed that facilitates weight loss [10,11]. Despite these benefits, the evidence of the causal relation between intake of PW and weight loss is not convincing [14].

The average PW consumption in Mexican adults aged >19 years in the year 2006 was 888 mL, which is at the lower end of the recommended intake for this population [15]. In the same group, the total beverage intake was 1721 mL [3], an amount below that recommended by the Institute of Medicine of the National Academies of the U.S.: 3.0 L for adult men and 2.2 L for adult women [16]. Several barriers to PW consumption have been identified among the Mexican population, including the limited infrastructure for its availability, poor hygiene of the public water distribution network, increase in the price of bottled water in recent years, marketing that promotes and facilitates the sale of CB and everyday knowledge that discourages its consumption [3,17-19].

According to official statistics 45.5% of Mexican population was classified as living under poverty conditions, and 9.8% were living in extreme poverty in 2012 [20]. In the same year, the national prevalence of overweight and obesity in adults was 71.3%. According to SES this prevalence was 65.6%, 72.7% and 73.5% in the low, medium and high SES, respectively [21]. Furthermore, the reported caloric intake from CB was 265, 307 and 306 kcal in the same low, medium and high SES, respectively [3]. Although the prevalence of overweight and obesity is lower in people with low SES, the trends observed in previous Health and Nutrition Surveys are towards an increase in growth velocity compared to the intermediate and high SES; for this reason we considered highly relevant the study of this group [22].

The association between PW intake and CB consumption in adults from the low SES in Mexico is unknown. This study is intended to contribute to its understanding. The suggested hypothesis is that subjects with a higher PW intake have a lower CB consumption. The objective of this study is to analyze the association among various levels of PW and CB consumption in low SES Mexican adults.

## Methods

### Design and population

Cross-sectional design. The information was collected in Cuernavaca, Mexico between March and October (2012). Cuernavaca is an urban area with more than 360,000 inhabitants located 85 km South of Mexico City. Mean, maximum and minimum atmospheric temperatures during this period were 23°C, 33°C and 14°C, respectively [23]. Sample selection was done using basic geographical areas (BGAs) of the municipality of Cuernavaca. During the first stage, BGAs were stratified by socioeconomic regions (low, medium and high) defined by the National Institute of Statistics and Geography of Mexico and those in low SES were selected (n = 6) [24]. During the second stage we selected neighborhoods evenly distributed in three areas: northern (n = 11), central (n = 12) and southern (n = 16). Finally, all the households from selected neighborhoods were visited by field personnel previously trained in the application of dietary and health questionnaires and standardized in anthropometric techniques [25]. Those households that did not respond during the first contact were visited once again outside of the work schedule. In order to identify the participants, a closed-ended questionnaire about health, comorbidities and some dinking and eating habits was utilized. Illiterate subjects and subjects with diarrhea, kidney or heart deficiency, urinary infections, or diabetes mellitus, or who habitually used diuretics or laxatives, and subjects suffering from anorexia, bulimia, who were pregnant or lactating or who had received nutritional counseling in the last 6 months, subjects with excessive alcohol consumption (>4 drinks or >1,420 mL/day for men, or >3 drinks or >1,065 mL/day for women) or who had a total beverage intake of >4 SD or >4 SD of PW, were excluded. Included were subjects of both sexes, between 21 and 59 years of age, with a body mass index (BMI) of >18.5 kg/m^2^. The study was reviewed and accepted by the research, ethics and biosafety committees of the National Institute of Public Health.

### Beverage consumption evaluation and classification

A 24-h beverage recall with the use of the U.S. Department of Agriculture five-step multiple-pass method was administered by an in-person interview to estimate beverage intake. This method consists of five steps: a) the quick list, which is a list of all beverages consumed in a 24-h period (midnight to midnight) the day prior to the interview; b) frequently forgotten beverages; c) a time and occasion, which queries the subject on the time, occasion and name of the beverages that have been consumed; d) the detail and review, which elicits descriptions of beverages and amounts consumed. In this section, a photo album with images of glasses, cups, mugs and bottles of various sizes that are frequently used for beverage consumption were used; e) the final probe, which asks respondents if anything else was consumed [26,27].

Estimation of the nutritional contribution of the beverages was made using the food and beverage nutritional composition tables of the National Institute of Public Health [28]. Those commercialized beverages for which no nutritional information was available were sought at supermarkets for the corresponding nutritional label; for beverages not found in these stores, the nutritional information of similar beverages in the nutritional composition tables of INSP was used. The beverages classification was based on a proposals by Popkin et al. and Rivera et al. This classification is based on health risk evidence according to beverages type and not necessarily on calories. The classification consists of six levels; level one is plain water. Levels two, three and four provide low energy and have some nutritional benefits. Whereas levels five and six provide the most energy and are related to negative health outcomes [15,29]. For this article the described levels have been regrouped on three mutually exclusive groups: 1) plain water; 2) caloric beverages (level five and six): sugar-sweetened beverages, sodas, whole milk, milk with added sugar, alcoholic beverages and fruit or vegetable juices; 3) low-fat milk, skim milk and beverages without added sugar (LFM-BWAS; levels two, three and four): low-fat milk, skim milk, sugar-free soy beverages, coffee and tea without sugar and light beverages (Additional file 1: Table S1).

### Classification of the population according to PW intake

Five groups were created based on PW intake: nondrinkers (ND), i.e., those who consumed no PW, plus four groups corresponding to quartiles of PW consumption (Q1, Q2, Q3, Q4). Q1 had an intake of 1 to ≤490 mL; Q2 >490 to <1,000 mL; Q3 ≥ 1,000 to ≥1,500 mL, and Q4 ≥ 1,500 mL.

### Anthropometry, physical activity, nutritional status, and covariables

The weight was measured on kg, with accuracy of 100 g with an electronic scale (SECA model 813). The height and waist circumference were measured on centimeters with a precision of 1 mm using a stadiometer (SECA Model 213) and a fiberglass tape (SECA model 201), respectively. These measurements were taken by standardized personnel according to Lohman’s methodology [25]. The presence of overweight or obesity was defined using the BMI and the classification of the World Health Organization [30]. Physical activity (PA) was measured using the short version of the International Physical Activity Questionnaire consisting of seven questions. It is easily applied and evaluates PA of the previous 7 days. It has been validated in adults and estimates the total metabolic equivalents utilized each day. PA was classified according to three levels of intensity (light, moderate and vigorous) [31]. Sociodemographic variables were obtained with a questionnaire that has been utilized in previous studies of the Mexican population.

### Statistical analysis

The arithmetic means for the continuous variables and proportions for the demographic, anthropometric, and PA variables were estimated to describe the analytic sample. In order to test the differences among the five PW intake groups, one-way ANOVA for quantitative variables and *χ*^2^ test for qualitative variables were calculated. Based on the classification of beverages, the CB/PW ratio was created in order to determine the CB intake in relation to the PW intake. Comparison of the intake of beverages among PW groups was adjusted with the Bonferroni test for multiple comparisons, and the Wilcoxon-type test for trend was calculated among the five groups to determine direction and significance of the intake of beverages in relation to PW intake [32].

A Heckman regression model was utilized for the dependent variable -CB- because the sample was regarded as truncated by the number of subjects with “zero” values (4%) in CB consumption. This problem skews the dependent variable and therefore the beta estimators obtained through the regression model. The Heckman model comprises two stages: in the first, a Probit model is calculated in order to obtain the selection probability (“lambda”) based on subjects who did and did not consume CB. The second stage consists of a linear regression model of minimum ordinal squares based on the truncated sample, adjusting the beta estimators for the selection probability obtained with the Probit model [33-35]. The assumptions of normality and linearity were previously evaluated by means of a linear regression model. Because the assumptions of constant variance of the residues were not met, the Heckman model was estimated with robust standard errors. A total of 1108 observations were included in the model, 42 of which were classified as truncated (ND n = 3; Q1 n = 4; Q2 n = 5; Q3 n = 7; Q4 n = 23). Based on the information of Pan et al. [36], we used sugary beverages as an approximate of caloric beverages. The mean intake of group 2 (144 ml/day) and group 5 (60 ml/day) was selected. The difference in the means between those two groups was 84 ml, which was used, along with a standard deviation of 265 ml for Q1 and 276 ml for Q4, to calculate the power of the study (93% with 95% confidence). In this way we were able to show a significant difference between Q1 and Q4 of CB intake. The analysis was performed with the STATA software v.12 [37]; p value <0.05 was considered statistically significant.

## Results

**Figure 1:**
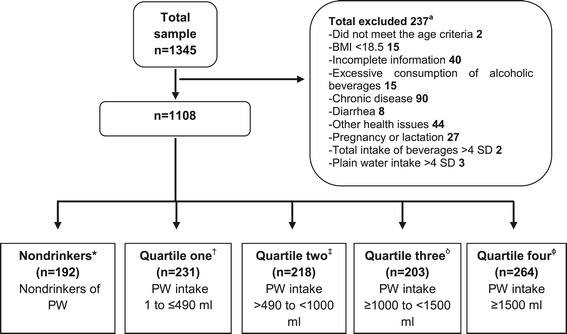
Flow chart of the study sample and population definition.

A total of 1345 subjects were evaluated and 237 were eliminated based on the exclusion criteria. A total of 1108 subjects −828 women (75%) and 280 men (25%)- with an average age of 37.7 ± 10 years were included in the analysis (Figure 1); other characteristics of the population may be observed in Table 1. In the final analytic sample, 17% were PW nondrinkers; 21% were located in Q1; 20% in Q2; 18% in Q3 and 24% in Q4.

**Table 1:** **Characteristics of adults with low SES according to PW intake**

		**Total (n = 1108)**		**Quartiles of PW intake**				**p value** ^*^
			**PW nondrinkers (n = 192)**	**One** ^**†**^ **(n = 231)**	**Two** ^**‡**^ **(n = 218)**	**Three** ^**δ**^ **(n = 203)**	**Four** ^**ϕ**^ **(n = 264)**	
**Sex [% (n)]**	Male	25 (280)	32 (62)	19 (44)	26 (56)	20 (40)	30 (78)	0.004
	Female	75 (828)	68 (130)	81 (187)	74 (162)	80 (163)	70 (186)	
**Age (mean ± SD)**	37.7 ± 10.2	37.3 ± 9.5	37.5 ± 9.5	36.9 ± 10.6	37.6 ± 10.0	38.7 ± 11.0	0.104	
**Education [% (n)]**	Elementary school and Junior High school	26 (291)	27 (52)	28 (66)	25 (55)	27 (55)	24 (63)	0.229
	High school	63 (698)	66 (126)	64 (147)	64 (137)	63 (127)	61 (161)	
	Graduate and postgraduate	11 (117)	7 (14)	8 (18)	11 (24)	10 (21)	15 (40)	
**BMI (mean ± SD)**	28.9 ± 5.1	28.3 ± 4.8	28.3 ± 4.6	28.7 ± 5.0	29.4 ± 5.7	29.6 ± 5.3	0.013	
	Normal [% (n)]	23 (251)	27 (51)	22 (52)	24 (54)	23 (46)	18 (48)	0.211
	Overweight [% (n)]	40 (442)	38 (74)	45 (105)	38 (82)	38 (77)	39 (103)	
	Obesity [% (n)]	37 (415)	35 (67)	32 (74)	38 (82)	39 (80)	43 (113)	
**WC** ^**γ**^ **(mean ± SD)**	93.98 ± 12.0	94.0 ± 12	92.6 ± 11.0	93.2 ± 11.6	95.3 ± 12.8	94.8 ± 12.52	0.184	
**Physical activity** ^**ε**^ **(mean ± SD)**	2805 ± 4300	2959 ± 4829	2425 ± 3250	3086 ± 4544	2446 ± 3895	3078 ± 4753	0.000	
	Light [% (n)]	26 (282)	29 (54)	24 (54)	24 (53)	30 (62)	22 (58)	0.351
	Moderate [% (n)]	47 (523)	45 (87)	51 (119)	46 (99)	46 (93)	47 (125)	
	Vigorous [% (n)]	27 (303)	26 (51)	25 (25)	30 (66)	24 (48)	31 (81)	

*ANOVA test for quantitative and *χ* ^2^ for qualitative variables among the five PW intake groups.

^γ^n = 1100.

^ε^MET-minutes/week of all levels of physical activity.

^†^1 to ≤490 mL; ^‡^ > 490 to <1000 mL; ^δ^ ≥ 1000 to <1500 mL; ^ϕ^≥ 1500 mL.

SES, socioeconomic status; WC, waist circumference; BMI, body mass index; PW, plain water.

The total average daily intake of beverages in the population was 2003 ± 870 mL. PW intake was 938 ± 816 mL; CB intake was 921 ± 598 mL and LFM-BWAS intake was 143 ± 297 mL. Men had a higher intake of beverages than women (2310 vs. 1899 mL of total beverages, 1020 vs. 910 mL of PW and 1137 vs. 848 mL of CB). A total of 21% of the intake (422 mL) was for sugar-sweetened beverages, and 15% (294 mL) was for sodas. Together these add up to more than one third of the total beverage consumption (36%).

In trend tests, a lower per capita CB consumption was observed to occur as PW intake increased (p <0.01) except for natural juices, the consumption of which increased (p <0.01), whereas the intake of whole milk and LFM-BWAD remained unchanged. The intake of sodas, whole milk and LFM-BWAD was similar among quartiles. Although there was a higher consumption of PW across quartiles, there were no differences in the average intake of these beverages among them (Table 2).

**Table 2:** **Beverage intake in adults with low SES according to PW intake**

	**Total** ^**†**^ **(n = 1108)**	**Nondrinkers of PW** ^**a,†**^ **(n = 192)**	**Quartiles of PW intake**				
			**One** ^**b,†**^ **(n = 231)**	**Two** ^**c,†**^ **(n = 218)**	**Three** ^**d,†**^ **(n = 203)**	**Four** ^**e,†**^ **(n = 264)**	P* value
**Plain water (mL)**	938 ± 816	0	336 ± 115	744 ± 149	1208 ± 159	2099 ± 596	
**CB (mL)**	921 ± 598	1222 ± 652^b,c,d,e^	948 ± 561^a,e^	956 ± 596^a,e^	804 ± 530^a^	741 ± 551^a,b,c^	<0.001
Sugar-sweetened beverages	422 ± 502	548 ± 577^d,e^	451 ± 495^e^	470 ± 528^e^	349 ± 436^a^	322 ± 447^a,b,c^	<0.001
Sodas	294 ± 362	456 ± 459^b,c,d,e^	297 ± 345^a^	260 ± 312^a^	253 ± 335^a^	234 ± 318^a^	<0.001
Whole and sweetened milk	163 ± 240	165 ± 249	156 ± 212	184 ± 240	176 ± 280	141 ± 223	0.263
Alcoholic beverages	22 ± 126	48 ± 184^d,e^	25 ± 135	24 ± 141	8.8 ± 336^a^	10 ± 77^a^	0.003
Natural juices	19 ± 98	4 ± 35^e^	19 ± 88	18 ± 93	17 ± 87	33 ± 140^a^	0.010
**Low-fat milk, skim milk and beverages without added sugar (mL)**	143 ± 297	206 ± 431^c,e^	145 ± 288	116 ± 234^a^	132 ± 241	126 ± 267^a^	0.445
Low calorie	132 ± 291	200 ± 431^c,e^	137 ± 281	107 ± 228^a^	121 ± 234	108 ± 251^a^	0.152
Low-fat milk	11 ± 68	6 ± 51	8 ± 47	10 ± 55	11 ± 71	18 ± 94	0.087
**Total beverages (mL)**	2003 ± 870	1427 ± 642^c,d,e^	1429 ± 570^c,d,e^	1818 ± 597^a,b,d,e^	2144 ± 540ª^,b,c,e^	2965 ± 760^a,b,c,d^	<0.001
**Energy from beverages (kcal)**	412 ± 321	537 ± 354^b,c,d,e^	423 ± 375^a,e^	418 ± 278^a^	366 ± 263^a^	342 ± 289^a,b^	<0.001
Ratio** (CB/PW)	1.43 ± 1.9	0	3.36 ± 2.8^c,d,e^	1.34 ± 0.9^b,d,e^	0.68 ± 0.5^b,c^	0.38 ± 0.3^b,c^	<0.001

^**†**^mean ± SD.

*Trend test value among the five groups.

^a,b,c,d,e^Difference between groups with p <0.05. Comparisons between groups were adjusted for Bonferroni correction.

**n = 916 due to the exclusion of 192 subjects who do not drink plain water.

SES, socioeconomic status; CB, caloric beverage; PW, plain water; CB/PW, ratio of caloric beverages to plain water.

Energy intake from beverages decreased as PW consumption increased (trend p <0.001). The largest difference was observed between groups with a lowest and a highest PW intake; ND vs. Q4 (195 kcal, p <0.001) and Q1 vs. Q4 (81 kcal, p <0.01). The mean CB/PW ratio was 1.43, i.e., 43% more CB than PW are consumed. A negative trend of the ratio between quartiles 1 and 4 was observed: 3.36, 1.34, 0.68 and 0.38, respectively (p <0.001, Table 2).

Heckman’s two-stage regression model showed that PW consumers had 0.5 less probability of consuming CB (p = 0.029) and that LFM-BWAS consumers were 0.9 less likely to consume CB (p <0.001). It was also observed that for every 100 mL of PW or LFM-BWAS ingested, CB intake decreased by 20 mL (p <0.001) and 47 mL (p <0.001), respectively, adjusting the regression model as required for PW or LFM-BWAS consumption and for age, sex, education, BMI, and PA (Table 3).

**Table 3:** **Probability and differences in consumption of CB according to PW intake in adults**

		**Coefficient**	**Standard error**	**Z**	**P**	**95% CI**
**First stage: Probit model**						
CB^**α**†^						
PW^**α**^		−0.5406	0.2482	2.18	0.029	−1.0271 – -0.0541
Low-fat milk, skim milk and beverages without added sugar (mL)^α^		−0.8721	0.1518	5.74	<0.001	−1.1698 – -0.5744
**Second stage: multiple linear regression model**						
CB (mL)^†^						
PW (mL)		−0.1924	0.0187	−10.24	<0.001	−0.2292 – -0.1555
Low-fat milk, skim milk and beverages without added sugar (mL)		−0.4673	0.0552	−8.46	<0.001	−0.5757 – -0.3590
Age (years)		−0.2708	1.6067	−0.17	0.8	−3.4199 – 2.8782
Sex (0 = male, 1 = female)		−279.5922	43.8127	−6.38	<0.001	−365.46 – -193.72
BMI (kg/m^2^)		6.2841	3.4234	1.84	0.066	−0.4257 – 12.9939
Education	1 = Elementary	1				
	2 = High school	108.9816	36.9371	2.95	0.003	36.586 – 181.377
	3 = Bachelor’s degree	257.6571	67.6873	3.81	<0.001	124.99 – 390.32
Physical activity	1 = Light	1				
	2 = Moderate	20.5962	37.7636	0.55	0.585	−53.41 – 94.611
	3 = Vigorous	80.8213	50.0588	1.61	0.106	−17.29 – 178.93
Constant		1392.072	135.28	10.29	<0.001	1126.91 – 1657.23

^**α**^0 = nondrinker; 1 = drinker.

^†^Dependent variable.

CB, caloric beverage; PW, plain water; BMI, body mass index.

## Discussion

In this population with low socioeconomic status, an inverse association was observed between PW and CB intake. There are few studies assessing the association between PW and CB intake. Consistent with our results, Popkin et al. observed, in a cross-sectional study representative of the U.S. population, that subjects aged >18 years who drink PW are less likely to consume sugar-sweetened beverages than PW nondrinkers [38]. Also, in a cohort study of adult women aged 25 to 42 years (Nurses’ Health Study), Pan et al. observed a negative correlation between PW and CB intake (r = 0.15) [36].

In the present study the mean difference in PW intake between quartiles 1 and 4 was >1.5 L; therefore, the difference in CB intake was more evident. These findings may be due to the fact that a higher PW intake covers a larger proportion of the fluid requirements, reduces feelings of thirst, and therefore the desire to consume CB [10,39]. Another potential reason for the observed reduction of CB intake in PW drinkers is confounding. PW intake has been observed to be associated with healthier lifestyles—including routine exercise and a higher consumption of vegetables, fruits and whole grain cereals which, in turn, are associated with a lower CB intake [38,39]. Body mass index is another important confounder due to underreporting of food and beverage intake by subjects with overweight or obesity [40]. In the present study we observed a trend to consume less CB as a consequence of PW intake using Heckman’s regression model adjusted by potential confounders (age, sex, BMI, education, PA and consumption of other beverages). Intake of sodas was not different among the PW consumption groups, i.e., PW consumption is not associated with a reduction of these beverages. Intake of sodas has been described not to depend directly on the hydration status, and its consumption by adults has been shown to be determined by social and hedonic causes such as consumption of alcoholic beverages, socialization with friends, family influences and consumption of fast food [41]. In Mexico, other elements, e.g., everyday knowledge and socialization practices have been described as additional determinants of soda consumption in adults with low SES [19]. Perhaps this explains in part why some interventions focused on reducing the consumption of regular soda through increased consumption of PW have failed to decrease their consumption [42]. On the other hand, a study integrating an educational component as part of its intervention that emphasized reduction of CB intake, and promoted consumption of PW demonstrated a decrease in the concentration of fasting glucose, an improvement in hydration (osmolarity of urine) and a decrease in energy intake from beverages. However, some of the findings were similar when study participants consumed diet beverages; for example weight loss after 6 months was similar between the PW and diet beverages groups with a decrease in 2 and 2.5% of total body weight, respectively [10].

A limitation of our study is a potential measurement bias due to the fact that the comorbidities were detected using a questionnaire in 35% of the sample and verbally in the remaining 65%. However, no differences (p >0.05) were found in the total consumption of beverages and PW between subjects who reported having had a comorbidity that affected their consumption of fluids and subjects without these comorbidities. It is therefore inferred that, if subjects with no verbal report of an illness had been included, there would have been no difference in the findings.

According to Hedrick et al. a “prudent” dietary pattern (which typically contains vegetables, fruits, legumes, whole grains, fish, and poultry) is positively associated to PW consumption and an occidental dietary pattern (typically contains red meat, processed meat, refined grains, sweets and dessert, french fries, and high-fat dairy products) is positively associated to CB intake [39,43]. However, we were not able to measure the intake of solid foods, and therefore to evaluate dietary patterns, which is a limitation in our study. Also, not having information on solid foods did not allow us to evaluate their contribution to total daily fluids which is typically between 20 and 30%. Nevertheless, beverage consumption is the main component of the daily supply of fluids (70–80%). Therefore, an analysis of their consumption requires focusing every effort on the attainment of a precise record of their intake. We consider that if solid foods were measured, the findings would have been similar but may have had less statistical significance because the consumption of solid foods contributes to the choice of beverages, as was noted before. Moreover results of the present study are not generalizable to all low SES Mexican population because the sampling strategy was not probabilistic and due to the exclusion criteria.

The main strength of the study is the use of the five-step multiple-pass method suggested by the U.S. Department of Agriculture to evaluate beverage intake. Its application was intended to avoid the memory bias as much as possible and increase the accuracy of the record. Although the use of a single evaluation makes it impossible to know the routine consumption of beverages, it allows the correct estimation of the arithmetic means that were utilized [44]. Another strength was the variability in the intake of beverages because there is a variety in the atmospheric temperature in the geographical area from which the subjects were selected, a fact that allows the observation of differences in the intake of beverages with respect to a broad range of PW intake. Finally, sample size was optimal to attain adequate statistical power to make inferences about CB intake.

This study supports the initiatives of the federal government in Mexico to reduce the consumption of CB and to increase PW intake among the Mexican population [15] by making PW more available and accessible [45] and by informing the population about the adverse effect of CB [15].

## Conclusions

Our study observed that a higher intake of PW is related with lower CB consumption; but no differences were found in the intake of sodas, dairy drinks and low-calorie beverages between quartiles of PW consumption, among Mexicans of low SES. This association suggests that future studies are warranted to determine if increasing PW intake in a low SES Mexican population can reduce intake of CB, an action that may result in potential public health benefits.

## Consent

Written informed consent was obtained from the patient for the publication of this report.

## Additional file

Additional file 1: Table S1. Beverage classification system.

## Abbreviations

PW: Plain water
CB: Caloric beverages
LFM-BWAS: Low-fat milk, skim milk and beverages without added sugar
SES: Socioeconomic status
BGAs: Basic geographical areas
BMI: Body mass index
ND: Nondrinkers
Q1: Quartile one
Q2: Quartile two
Q3: Quartile three
Q4: Quartile four
PA: Physical activity
