# Supplementary material for: Plain water consumption is associated with lower intake of caloric beverage: cross-sectional study in Mexican adults with low socioeconomic status
Source: BMC Public Health. 2015 Apr 19;15:405. doi: 10.1186/s12889-015-1699-0 (PMC4411745; doi:10.1186/s12889-015-1699-0)
Supplement: Additional file 1: Table S1. — Beverage classification system. [file 12889_2015_1699_MOESM1_ESM.docx]

**Preparing additional files**

**Supplemental Table 1. Beverage classification system**

| **Classification** | **Group** | **Beverages** |
| --- | --- | --- |
| PW | PW | Plain water, mineral water, filtered or unfiltered tap water. |
| CB | Sugar-sweetened beverages | Fresh fruit beverages, all flavors (orange, lime, rice, hibiscus, etc.); Commercialized fruit juice; drink mixes of flavored powder and sugar; sports drinks; corn, rice, oats or chickpea *atole*; coffee and tea, soy beverages, energizing beverages and other sugar-sweetened beverages |
|  | Soda | Soda, all flavors |
|  | Whole milk and sweetened milk | Whole milk, classic and liquid yogurt, flavored milk (chocolate, vanilla, strawberry, etc.), sweetened milk, smoothies and fruit milkshakes (with or without cereal), coffee or tea with milk (with or without sugar), cappuccino frappé (mocha, latte, etc.) |
|  | Alcoholic beverages | Beer, alcohol-free beer, red wine, white wine, vodka, whiskey, gin, tequila, *pulque*, eggnog and *tepache* |
|  | Natural juices | Fruit and/or vegetable juices: all fruits and vegetables |
| Low-fat milk, skim milk and beverages without added sugar | Low-calorie | Coffee and tea without sugar or with non-caloric sweetener, water with chlorophyll, zero type or light sodas, water with non-caloric powdered drink mix, light juices, light teas |
|  | Low-fat milk | Skim milk, light milk, sugar-free coffee or tea with low-fat milk and sugar-free soy beverages |

PW, plain water; CB, caloric beverage.
